# Islet-autoreactive CD4^+^ T cells are linked with response to alefacept in type 1 diabetes

**DOI:** 10.1172/jci.insight.167881

**Published:** 2023-11-08

**Authors:** Elisa Balmas, Janice Chen, Alex K. Hu, Hannah A. DeBerg, Mario G. Rosasco, Vivian H. Gersuk, Elisavet Serti, Cate Speake, Carla J. Greenbaum, Gerald T. Nepom, Peter S. Linsley, Karen Cerosaletti

**Affiliations:** 1 Center for Translational Immunology, and; 2 Center for Systems Immunology, Benaroya Research Institute, Seattle, Washington.; 3 Immune Tolerance Network, Bethesda, Maryland, USA.; 4 Center for Interventional Immunology and Diabetes Clinical Research Program, Benaroya Research Institute, Seattle, Washington, USA.

**Keywords:** Autoimmunity, Diabetes, Immunotherapy, T cells

## Abstract

Variation in the preservation of β cell function in clinical trials in type 1 diabetes (T1D) has emphasized the need to define biomarkers to predict treatment response. The T1DAL trial targeted T cells with alefacept (LFA-3–Ig) and demonstrated C-peptide preservation in approximately 30% of new-onset T1D individuals. We analyzed islet antigen–reactive (IAR) CD4^+^ T cells in PBMC samples collected prior to treatment from alefacept- and placebo-treated individuals using flow cytometry and single-cell RNA sequencing. IAR CD4^+^ T cells at baseline had heterogeneous phenotypes. Transcript profiles formed phenotypic clusters of cells along a trajectory based on increasing maturation and activation, and T cell receptor (TCR) chains showed clonal expansion. Notably, the frequency of IAR CD4^+^ T cells with a memory phenotype and a unique transcript profile (cluster 3) were inversely correlated with C-peptide preservation in alefacept-treated, but not placebo-treated, individuals. Cluster 3 cells had a proinflammatory phenotype characterized by expression of the transcription factor BHLHE40 and the cytokines GM-CSF and TNF-α, and shared TCR chains with effector memory–like clusters. Our results suggest IAR CD4^+^ T cells as a potential baseline biomarker of response to therapies targeting the CD2 pathway and warrant investigation for other T cell–related therapies.

## Graphical abstract

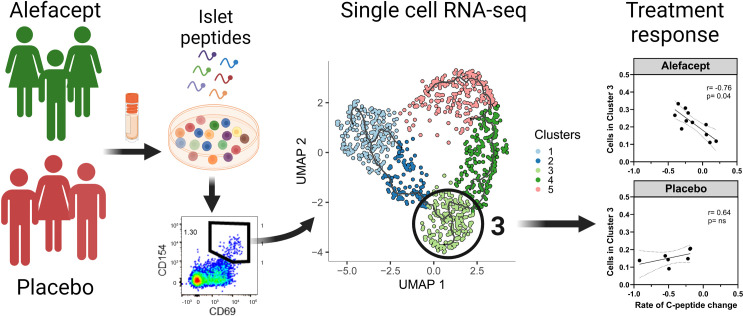

## Introduction

Type 1 diabetes (T1D) is an autoimmune disease leading to the destruction of pancreatic β cells and consequently to lifelong dependence on insulin. β Cells are silently destroyed (1) during a period of preclinical autoimmunity, which varies in length among individuals, and is characterized by an accumulation of autoantibodies against β cell antigens (2) and the appearance of islet-autoreactive T cells in the periphery and in the tissue (3, 4). The ultimate clinical goal is to predict which individuals will develop disease and intervene therapeutically to block the islet autoimmune response and preserve insulin secretion during the preclinical period. Also, a key clinical goal is to predict response to therapy prior to treatment to stratify at-risk and T1D patients to the most effective interventions or dosing, so-called personalized medicine.

Clinical trials targeting T cells in new-onset T1D patients have demonstrated transient preservation of β cell function (5–11), albeit with variability in the response to therapy. One example is the T1DAL trial of alefacept, a lymphocyte function–associated antigen 3–Ig (LFA-3–Ig) fusion protein that binds the costimulatory molecule CD2 (12) on memory T cells and NK cells. Mechanistically, alefacept disrupts CD58-mediated costimulation of T cells (13), and selectively depletes memory and effector T cells (14, 15) via NK-mediated antibody-mediated cytotoxicity (16, 17). In the T1DAL trial, alefacept treatment resulted in significant preservation of endogenous insulin production in approximately 30% of treated individuals (responders) after 2 years compared with placebo-treated participants (18, 19). Alefacept treatment in the responders depleted CD4^+^ effector memory and central memory T cells (TEM and TCM cells, respectively) while preserving regulatory T cells (Tregs), and preservation of insulin C-peptide was associated with the development of 2 CD8^+^ memory T cell populations with exhaustion-like features (20).

The variability in response to alefacept in different patients highlights the need for biomarkers that will predict response to treatment. One study has reported that a higher frequency of antiinflammatory CD4^+^CD25^+^CD127^hi^ T cells at diagnosis is positively correlated with a favorable response to alefacept (21). Here, we investigate autoreactive CD4^+^ T cells specific for epitopes in islet proteins as potential biomarkers that at baseline predict response to alefacept in new-onset T1D patients enrolled in the T1DAL clinical trial. Previous studies from our laboratory used single-cell RNA sequencing (scRNA-seq) to identify unique features of rare islet antigen–reactive (IAR) CD4^+^ T cells in T1D by capturing the T cell receptor (TCR) chains in parallel with the transcriptome of individual IAR memory T cells (22, 23). We observed that some IAR memory CD4^+^ T cells were clonally expanded in the peripheral blood of T1D patients and that expanded T cells had distinctive transcript phenotypes compared with nonexpanded islet T cells and had increased sharing of TCR α chains (22, 23). In this current study, using flow cytometry and scRNA-seq, we investigated IAR CD4^+^ T cells in pretreatment peripheral blood from T1DAL participants with the goal of identifying biomarkers of response to alefacept prior to treatment (18, 19). Analysis identified a subset of IAR CD4^+^ T cells with a memory phenotype and a unique transcript profile characterized by the expression of the transcription factor BHLHE40 and increased production of proinflammatory cytokines that correlated with poor response to treatment with alefacept.

## Results

### IAR CD4^+^ T cells in new-onset T1D patients have diverse phenotypes.

We set out to assess the cell surface phenotype of IAR CD4^+^ T cells in peripheral blood mononuclear cells (PBMCs) collected prior to treatment from 11 alefacept- and 7 placebo-treated new-onset T1D patients enrolled in the T1DAL clinical trial (Table 1). Participants in the current study were selected to have a broad range of change in C-peptide levels (calculated as the rate of change in 2-hour C-peptide AUC) over the course of the clinical trial, a surrogate indicator of insulin secretion (Table 1). They ranged in age from 12 to 32 years and were 44% female. All participants carried at least 1 copy of one of the T1D high-risk HLA class II alleles, DRB1*04, DRB1*03, or DQB1*03; 15 participants carried DR4 only, 3 were DR3/DR4, and 5 were DR3 only.

**Figure 1:**
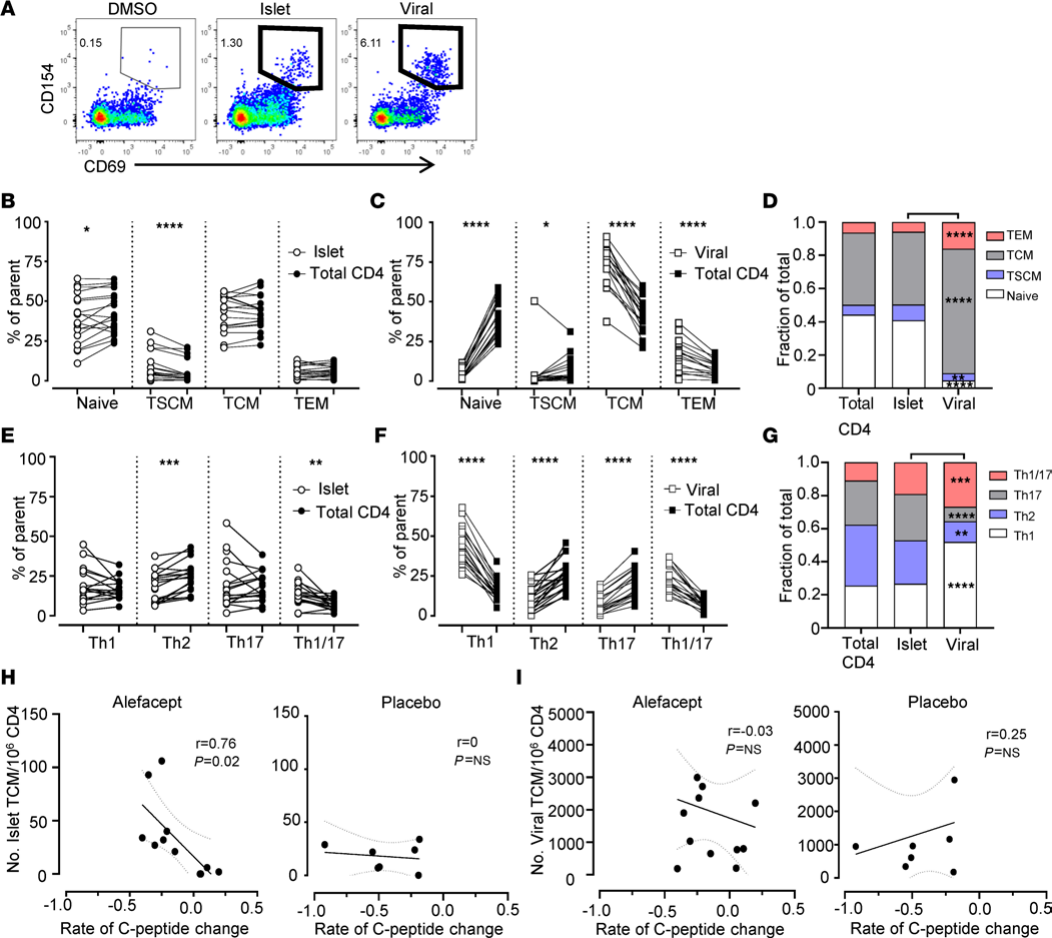
IAR CD4^+^ T cells have heterogeneous phenotypes and correlate with alefacept response. (**A**) Antigen-reactive CD4^+^ T cells were gated as CD154^+^CD69^+^ based on the DMSO vehicle control (participant T1DAL-243767). (**B** and **C**) The frequency of naive and memory populations in IAR and viral antigen–reactive CD4^+^ T cells in baseline PBMC samples from the treated and placebo groups (*n* = 18). Enriched antigen-reactive cells were compared with total CD4^+^ T cell populations from the pre-enrichment samples of the same cultures. Stem cell memory (TSCM), central memory (TCM), and effector memory (TEM) are shown as the percentage of antigen-reactive or of total CD4^+^ T cells; each symbol represents a unique individual. (**D**) The mean frequency of each population from **B** and **C**. Asterisks indicate significant differences between IAR and viral antigen–reactive populations. (**E** and **F**) The frequencies of enriched IAR and virus-reactive memory CD4^+^ T cells with the indicated T helper phenotypes versus CD4^+^ T cells from the pre-enrichment samples of the same cultures (*n* = 18). Th1 (CXCR3^+^CCR4^–^CCR6^–^), Th2 (CCR4^+^CCR6^–^), Th17 (CCR6^+^CCR4^+^), and Th1/17 (CXCR3^+^CCR6^+^CCR4^–^) are expressed as the frequency of memory antigen-reactive CD4^+^ T cells or total memory CD4^+^ T cells. (**G**) The mean frequency of each Th subset from **E** and **F**. Asterisks indicate significant differences between IAR and viral antigen–reactive populations. Significant differences in **B**–**G** were determined using Wilcoxon’s matched-pairs signed-rank test with Benjamini-Hochberg adjustment. *P < 0.05; **P < 0.01; ***P < 0.001; ****P < 0.0001. (**H** and **I**) Spearman’s correlation between the frequency of IAR CD4^+^ TCM cells (**H**) or virus-reactive TCM cells (**I**) per participant with the rate of C-peptide change in alefacept- and placebo-treated participants. The linear regression line is shown with 95% confidence intervals in dotted lines.

We performed an overnight activation-induced marker assay to identify IAR CD4^+^ T cells by the expression of the activation marker CD154 (22, 23). Banked PBMCs from the baseline visit were stimulated with a pool of 35 peptides from the islet proteins GAD65 (glutamate decarboxylase 2, 65 kDa isoform), IGRP (glucose-6-phosphatase 2 isoform 1), ZnT8 (zinc transporter 8 isoform a), IA-2 (islet cell antigen 512, protein tyrosine phosphatase receptor type N), PPI (preproinsulin), and Ins B (insulin B) that comprise immunodominant epitopes recognized by CD4^+^ T cells in T1D patients in the context of HLA DRB1*0401, DRB1*0301, and DQ8 (Supplemental Table 1; supplemental material available online with this article; https://doi.org/10.1172/jci.insight.167881DS1). As controls, PBMCs were stimulated with vehicle alone or a pool of viral peptides from cytomegalovirus, adenovirus 5, and influenza A virus. Activated CD154^+^ cells were enriched and analyzed by flow cytometry to identify CD4^+^CD154^+^CD69^+^ islet and viral antigen–reactive T cells (Figure 1A). IAR CD4^+^ T cells were single-cell sorted for subsequent scRNA-seq analysis. There was no significant difference detected in the frequency of IAR CD4^+^ T cells or viral antigen–reactive T cells between alefacept- and placebo-treated participants (Supplemental Figure 2A).

First, we explored whether IAR CD4^+^ T cells differed in maturation or T helper cell polarization compared with total CD4^+^ T cells or virus-reactive T cells. IAR T cells were heterogeneous in phenotype, representing all naive and memory phenotypes, compared with virus-reactive, which were all memory in phenotype (*P* < 0.05 to *P* < 0.0001, Supplemental Figure 2B). The majority of IAR CD4^+^ T cells were naive and TCM in phenotype, in similar proportions as detected in total CD4^+^ T cells from the same cultures (Figure 1, B and D). In contrast, viral antigen–reactive T cells from the same participants were exclusively TCM and TEM in phenotype and differed significantly from frequencies observed in total CD4^+^ T cells (*P* < 0.05 to *P* < 0.0001) (Figure 1, C and D, and Supplemental Figure 2B). Notably, IAR CD4^+^ T cells had a significantly increased frequency of cells with a stem cell memory T (TSCM) cell phenotype compared with total CD4^+^ or virus-reactive CD4^+^ T cells (*P* < 0.0001 and *P* < 0.05, respectively). All Th subsets were present among IAR CD4^+^ T cells, with similar frequencies of cells with a Th2 phenotype as more pathogenic Th1, Th17, and Th1/17 phenotypes (Figure 1, E and G). Compared with the total CD4^+^ population, IAR T cells had significantly fewer cells with a Th2 phenotype (*P* < 0.001) but a significant increase in Th1/17 phenotype (*P* < 0.01). By contrast, virus-reactive T cells were primarily Th1 and Th1/17 polarized compared with IAR CD4^+^ T cells (*P* < 0.001), while IAR T cells had significantly higher frequencies of cells with a Th2 (*P* < 0.01) and Th17 phenotype (*P* < 0.001) compared with virus-reactive T cells (Figure 1, F and G, and Supplemental Figure 2C).

Expression of individual surface markers confirmed that IAR CD4^+^ T cells expressed CD2, the target of alefacept. CD2 was expressed on greater than 95% of IAR CD4^+^ T cells, comparable to total CD4^+^ T cells (Supplemental Figure 2D). The levels of CD2 expressed on IAR CD4^+^ T cells were also significantly higher than those detected on total CD4^+^ cells (*P* < 0.001), as measured by mean fluorescence intensity (MFI). Compared with virus-reactive T cells, IAR CD4^+^ T cells were less CD2 positive (*P* < 0.001) and expressed lower CD2 levels than was detected on virus-reactive T cells (Supplemental Figure 2, D and F). Increased CD2 levels in IAR and virus-reactive CD4^+^ T cells compared with total CD4^+^ T cells were likely due to overnight activation with peptide; the higher avidity for the foreign antigen in T cell activation likely mediated greater upregulation of CD2 on virus-reactive T cells than was detected on IAR CD4^+^ T cells.

Consistent with increased Th1/17 cells, IAR T cells were more CXCR3 positive and CCR6 positive than total CD4^+^ T cells (*P* < 0.01) and did not differ from virus-reactive T cells (Supplemental Figure 2, E and F). Expression of PD-1 was also increased on IAR CD4^+^ T cells compared with total CD4^+^ T cells (*P* < 0.0001), reflecting activation in the CD154 assay, but fewer IAR CD4^+^ T cells expressed PD-1 and TIGIT than virus-reactive cells (Supplemental Figure 2, E and F). Interestingly, CD38 expression on IAR CD4^+^ T cells did not differ from total CD4^+^ T cells but was significantly increased compared with virus-reactive cells (*P* < 0.0001) (Supplemental Figure 2F). The increase in CD38^+^ IAR T cells was limited to the TSCM and TCM populations (Supplemental Figure 2G).

Lastly, we determined whether the frequency of IAR CD4^+^ T cells with a particular phenotype was linked to the rate of change in C-peptide levels in the alefacept versus placebo groups. The number of total IAR CD4^+^ T cells (*r* = –0.80, *P* = 0.02) and specifically IAR T cells with a TCM phenotype (*r* = –0.76, *P* = 0.02) were significantly correlated with C-peptide decline in the alefacept- but not placebo-treated group (Figure 1H and Supplemental Figure 2H). There was no correlation of virus-reactive CD4^+^ T cells with C-peptide decline in the alefacept- or placebo-treated groups, indicating the correlation with C-peptide decline in alefacept-treated participants was specific for IAR CD4^+^ T cells (Figure 1I and Supplemental Figure 2I). We also detected a significant correlation of IAR CD4^+^ T cells in the alefacept group with the quantitative response (QR), which adjusts C-peptide levels at 12 months for age and baseline C-peptide (data not shown and ref. 24). There was no correlation detected between IAR CD4^+^ T cells and QR in the placebo group. We did not detect any significant association of C-peptide decline with responder status (data not shown), IAR CD4^+^ T cell Th lineage, or CD2 expression on IAR CD4^+^ T cells (Supplemental Figure 2, J and K).

### IAR CD4^+^ T cell transcript profiles form a trajectory based on maturation and activation.

**Figure 2:**
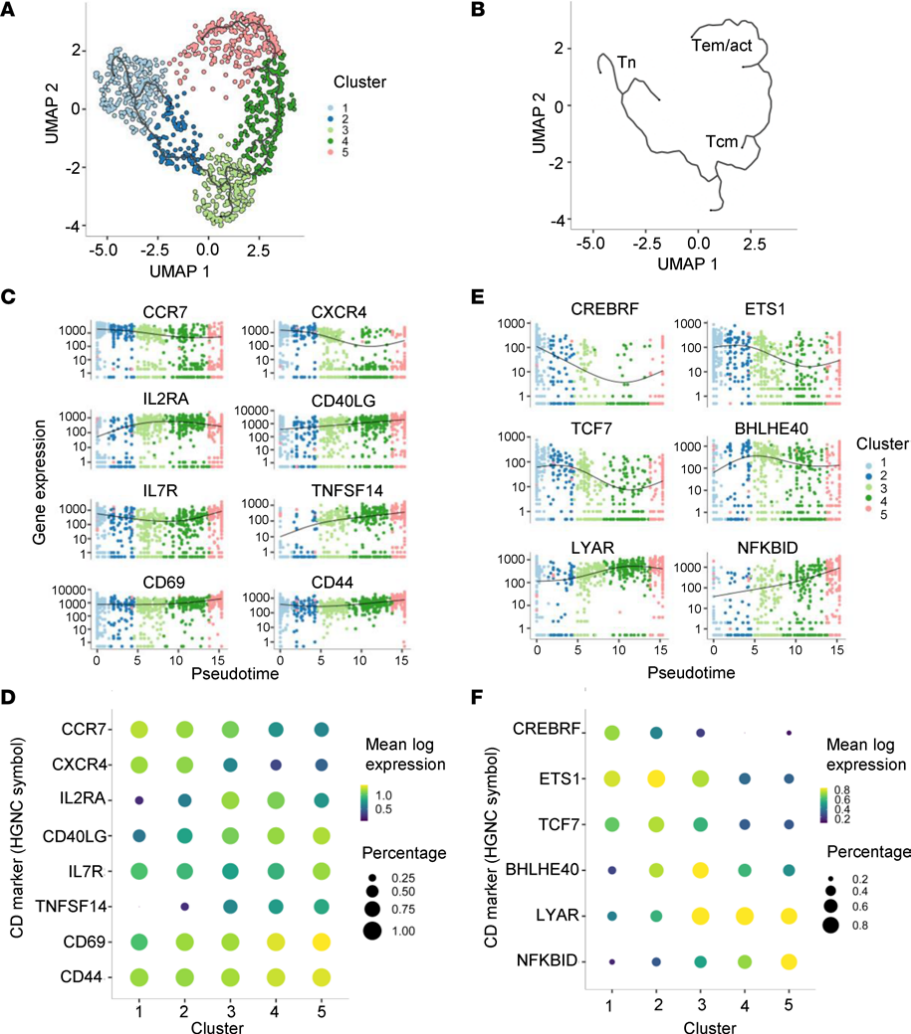
scRNA-seq profiles from IAR CD4^+^ T cells form a trajectory following differentiation and activation. (**A**) UMAP plot of Leiden clustering of scRNA-seq profiles of IAR CD4^+^ T cells (*n* = 1,014 cells) from T1DAL participants (*n* = 18) defines 5 clusters of cells with unique phenotypes (Supplemental Table 3). Each symbol represents an individual cell from a study participant. The black line denotes a trajectory graph calculated using Monocle 3. (**B**) Monocle 3 trajectory graph depicted without cells to show inferred transcriptome phenotypes of IAR CD4^+^ T cells: naive (Tn), central memory (Tcm), effector memory (Tem), and activated (act) T cells. (**C**) Pseudotime plots (Monocle 3) of indicated marker transcript levels (log_10_ transformed) versus clusters. Genes were defined by the top_marker function of Monocle 3. (**D**) Bubble plot of marker genes in **C**. The color scale indicates mean log expression level of each gene, and the size of each circle indicates the percentage of cells in the indicated cluster that express the gene according to the legend. (**E**) Pseudotime plots of transcript levels for the indicated transcription factor genes versus clusters. (**F**) Bubble plot of transcription factors in **E**.

To further characterize the phenotypic heterogeneity of IAR CD4^+^ T cells, we analyzed the scRNA-seq transcript profiles from CD154^+^CD69^+^ cells using the Monocle 3 toolkit (25) to cluster cells along a pseudotime trajectory. Pseudotime orders an asynchronous population of cells along a learned trajectory based on their gene expression, reflecting progress through different cell states, such as differentiation. IAR CD4^+^ T cells from all participants (*n* = 1,014 cells) formed a relatively continuous trajectory consisting of 5 clusters of cells, as shown in the uniform manifold approximation and projection (UMAP) dimensionality reduction plot in Figure 2A. To maximize the reproducibility of clustering in Monocle 3, we set a seed for the pseudorandom number generator. We also ensured reproducibility by repeating the clustering multiple times. Finally, we confirmed that Monocle 3 clusters included IAR CD4^+^ T cells from all participants, except for cluster 2 which lacked cells from participant T1DAL-323347 (alefacept group), and that none of the clusters were dominated by sample bias or sample-specific characteristics (Supplemental Figure 3, A–D, and Supplemental Table 3). IAR CD4^+^ T cells were distributed evenly across the Monocle clusters apart from cluster 2, which had significantly fewer cells than the other clusters (Supplemental Figure 3C).

Cell clusters were annotated by mapping reference PBMC populations to the IAR CD4^+^ T cell trajectory using Seurat, which indicated that the trajectory reflected the maturation and activation characteristics of the cells (Figure 2B). The top marker function in Monocle was used to identify expression of genes enriched in each cluster, including surface proteins (Figure 2, C and D) and transcription factors (Figure 2, E and F). Thus, clusters 1 and 2 were composed of naive-like IAR CD4^+^ T cells with higher expression of the chemokine receptor genes *CCR7* and *CXCR4* and transcription factor *TCF7*, and a lower level of activation based on expression of *CD40LG* (CD154), *CD69*, *CD44*, and *TNFSF9* (CD137) (Figure 2, C and D, and Supplemental Figure 3C). Cluster 3 was composed of TCM-like cells with expression of *CCR7*, and increased expression of *IL2RA* and the transcription factor *BHLHE40*. Clusters 4 and 5 were composed of TEM-like cells characterized by low expression of *CCR7*, *CXCR4*, *TCF7*, and *CREBRF*, but higher expression of *LYAR* and *NFKBID*. Notably, clusters 3, 4, and 5 showed a gradient of increasing activation based on expression of *CD40LG* (CD154), as well as *CD69* and *CD44* (Figure 2D).

**Figure 3:**
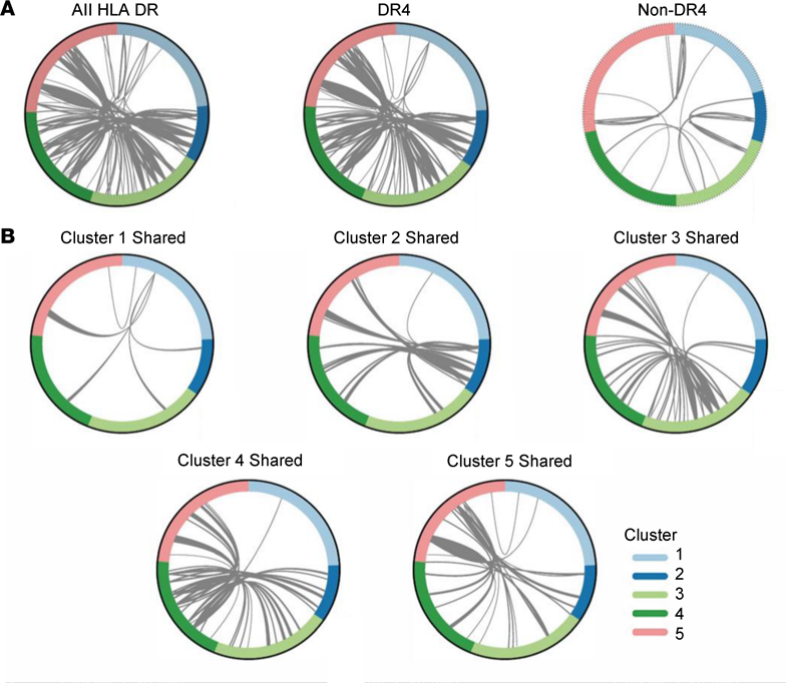
Expanded IAR CD4^+^ T cells share TCRs between clusters with memory transcript profiles. (**A**) Circos plots showing TCR chain junction (V-junction-J) nucleotide sequences shared between ≥2 IAR CD4^+^ T cells within or between clusters for all 18 participants. Plots depict sharing between cells regardless of donor HLA (all HLA DR: 993 total cells with 1,954 productive TCR chains, 122 cells with shared chains, 44 unique chains), between cells from 13 individuals carrying a DRB1*04 allele (DR4: 776 total cells with 1,535 productive TCR chains, 102 cells with shared chains), or between cells from 5 individuals with no DRB1*0401 allele (non-DR4: 217 total cells with 419 productive TCR chains, 20 cells with shared chains). Each segment in the outer circle represents an individual IAR CD4^+^ T cell with a TCR chain colored by cluster, as indicated in the legend. Arcs connect cells that share identical *TRA* and/or *TRB* chains; line thickness corresponds to the number of chains shared between each cell. In DR4 individuals there were 71 cells with 2 shared chains (primarily *TRA*-*TRB* pairs), 22 cells that shared 1 chain (*TRB* > *TRA*), and 9 cells sharing >2 chains per cell. Of the expanded cells, 88 shared TCR chains within donors (private) and 12 were shared between donors (public). (**B**) Circos plots as in **A** showing TCR chains shared between cells in clusters 1–5 in DR4 individuals. Each plot represents TCR chains in cells from an individual cluster that are shared with cells in other clusters, as indicated by the arcs connecting cells between clusters. Expanded cells comprised approximately 2%, 14%, 26%, 23%, and 35% of cells in clusters 1–5, respectively.

Our previous studies demonstrated expansion of IAR CD4^+^ T cells in individuals with T1D (22, 23). To assess the clonal relatedness of IAR CD4^+^ T cells along and across the Monocle pseudotime trajectory, we identified TCR chains sharing junction nucleotide sequences between 2 or more cells (expanded cells). We first compared sharing between all TCR chains regardless of HLA type (18 individuals) and identified 122 expanded cells from 16 out of 18 participants in both the alefacept and placebo groups that shared 44 unique TCR chains (Figure 3A). The majority of expanded cells shared both TCR α (*TRA*) and β (*TRB*) chains, followed by sharing of only a single *TRA* or *TRB* chain (*TRB* > *TRA*), and a few cases of sharing of 3 chains. Sharing was detected predominantly within IAR CD4^+^ T cells of individuals (15 participants) rather than between individuals (5 participants), reflecting greater numbers of private versus public TCRs in this data set (23). We next analyzed TCR sharing in relation to HLA to avoid bias in estimating sharing. DR4-positive individuals (*n* = 13) were defined as those having at least one HLA-DRB1*04 allele and were compared to DR4-negative individuals (*n* = 5) (Figure 3A). This analysis showed TCR chain sharing between IAR CD4^+^ T cells in both DR4 and non-DR4 individuals, with more sharing among DR4-positive individuals than DR4-negative individuals, reflecting both the greater number of DR4-positive individuals tested and the prevalence of DR4-restricted peptides (*n* = 29) versus non–DR4-restricted peptides (*n* = 6) in the peptide pool used for stimulation. Considered as a percentage of total TCRs tested, we observed similar percentages of expanded TCRs in IAR CD4^+^ T cells from DR4-positive versus DR4-negative individuals (79% versus 84%, respectively).

Expanded IAR CD4^+^ T cells from all clusters of DR4-positive individuals shared junctions with cells in other clusters (Figure 3, A and B). Cells sharing identical TCR junction nucleotide sequences in different transcriptome clusters indicated heterogeneous expression profiles between clonally related cells. Expanded cells comprised approximately 2%, 14%, 26%, 23%, and 35% of cells in clusters 1–5, respectively (Figure 3B). The distribution between clusters for cells with expanded junctions differed from the distribution between clusters of total cells (*P* = 0.0079, Kolmogorov-Smirnov test), with cluster 1 and cluster 2 having proportionally less sharing, and clusters 3–5 having more shared TCR chains. This supports the distribution of T cells from naive to memory/effector/activated phenotypes along the proposed trajectory (Figure 2B), with more naive cells showing less sharing (i.e., less expansion). Although our studies were underpowered for analysis of junction amino acid sequence motifs, we did not note any obvious patterns of overrepresentation by visual analysis.

Four expanded TCRs from this study were previously shown to be specific for islet epitopes from GAD65 and ZnT8 (23). We also compared expanded TCR chain junction amino acid sequences to databases of TCRs of known specificities, VDJbd (26) and McPAS (27), which identified 10 out of 44 chains (6 *TRA* junctions, 4 *TRB* junctions) that matched a single chain from TCRs reported to recognize microbial or dietary antigens, including epitopes from EBV, CMV, HIV-1, influenza A, and *Mycobacterium tuberculosis*. One *TRA* junction matched a *TRA* chain from a celiac disease TCR recognizing the immunodominant epitope DQ2.5-glia-α2 (28). None of these matches included both the *TRA* and *TRB* chains of a single TCR and most were not 3-point matches encompassing the V gene, CDR3 sequence, and J gene.

### IAR CD4^+^ T cells with a cluster 3 phenotype are linked with response to alefacept.

**Figure 4:**
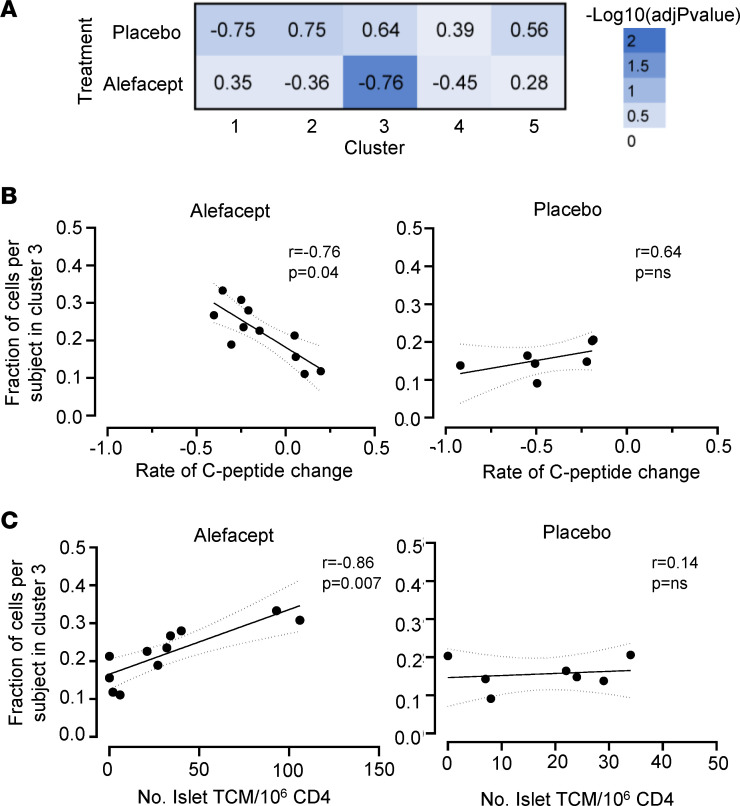
The frequency of IAR CD4^+^ T cells with a cluster 3 phenotype is linked with C-peptide change in alefacept-treated new-onset T1D patients. (**A**) Heatmap representation of adjusted *P* values from Spearman’s correlations of the fraction of IAR CD4^+^ T cells per individual in each Monocle cluster versus the rate of C-peptide change in alefacept-treated (*n* = 11) and placebo-treated (*n* = 7) participants over the 2-year clinical trial, as listed in Table 1. Spearman’s *r* values are shown in each square. (**B**) Correlation between the fraction of IAR CD4^+^ T cells per individual in cluster 3, with rate of C-peptide change in alefacept- and placebo-treated individuals performed as in **A**. The linear regression line is shown with 95% confidence intervals in dotted lines. (**C**) Spearman’s correlation of the fraction of IAR CD4^+^ T cells subject in Monocle cluster 3 versus the frequency of IAR CD4^+^ TCM cells per 1 × 10^6^ CD4^+^ T cells in PBMCs from alefacept- (*n* = 11) or placebo-treated (*n* = 7) individuals. *P* values were adjusted for multiple testing using the Benjamini-Hochberg test correction.

To determine whether IAR CD4^+^ T cells with a particular transcript phenotype were associated with response to therapy, we compared the distribution of cells across the 5 Monocle clusters with C-peptide change in each participant in the alefacept and placebo groups (Figure 4A). This analysis showed that the fraction of cells in cluster 3 from each participant was inversely correlated with the rate of C-peptide change (*r* = –0.76, *P* = 0.04) in the treatment group but not in the placebo group (*r* = 0.64, *P* = NS) (Figure 4B). No other Monocle clusters were significantly correlated with the rate of C-peptide change in the alefacept or placebo group (Figure 4A). Thus, alefacept-treated individuals who had a higher fraction of IAR CD4^+^ T cells with a cluster 3 transcript profile at baseline experienced a greater decline in C-peptide over the course of the clinical trial compared with those with a lower percentage of cluster 3 cells.

The relationship between cluster 3 cells and C-peptide change mirrored that of IAR CD4^+^ TCM cells (Figure 1H). To determine whether cluster 3 cells and IAR CD4^+^ TCM cells were directly related, we correlated the frequency of IAR CD4^+^ TCM cells per individual with the fraction of cells per individual in cluster 3 (Figure 4C). We detected a significant direct correlation between cluster 3 cells and IAR CD4^+^ TCM cells in alefacept-treated participants (*r* = 0.86, *P* = 0.007), but not in the placebo group (*r* = 0.14, *P* = NS), suggesting cells with a cluster 3 transcript phenotype contributed to the association of IAR CD4^+^ TCM cells with alefacept response. No other clusters were correlated with the frequency of IAR TCM cells.

### Cluster 3 cells have a proinflammatory phenotype.

**Figure 5:**
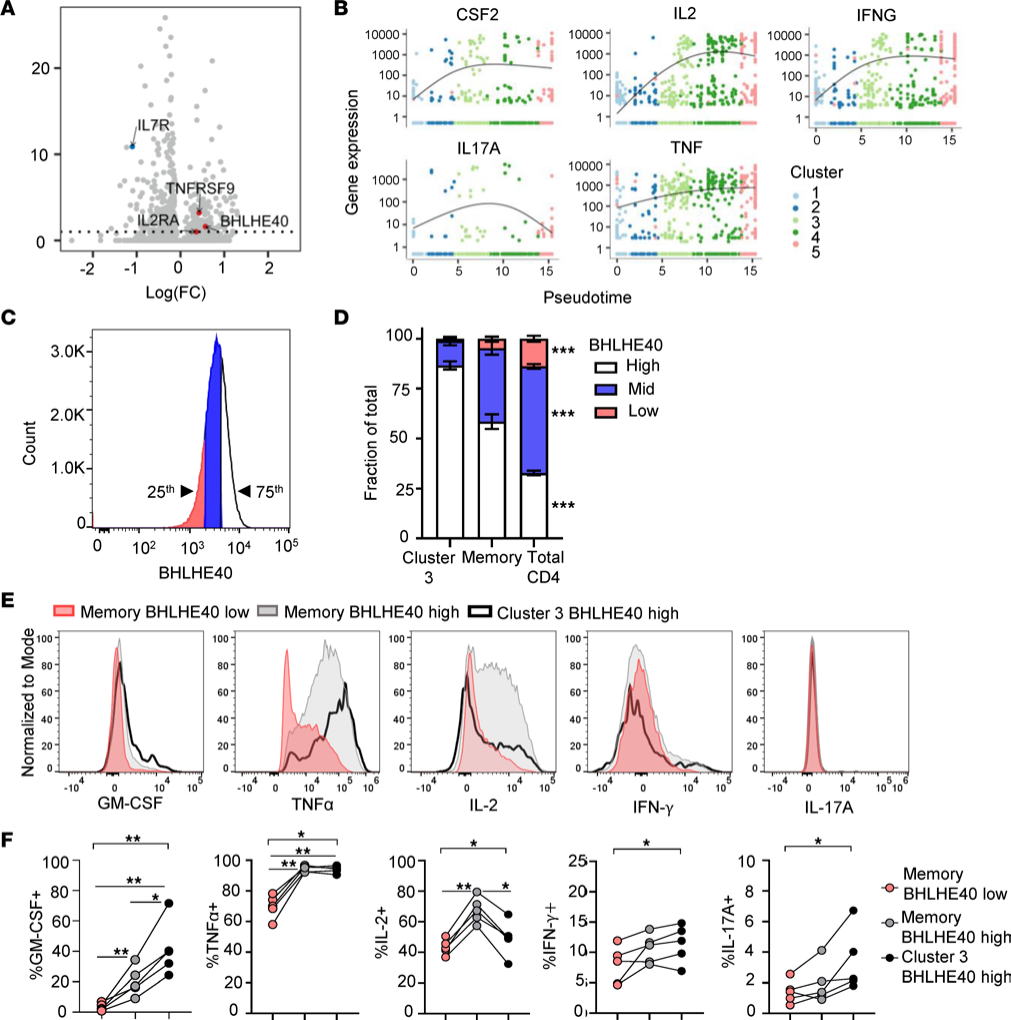
Cluster 3 IAR CD4^+^ T cells have a proinflammatory phenotype. (**A**) Volcano plot showing –log_10_-adjusted FDR vs. log(fold change) [log(FC)] for genes differentially expressed between cluster 3 cells and all other clusters, as determined by the fit_models linear regression function in Monocle 3. The dashed line denotes an adjusted *P* value of 0.05. Red dots, selected genes with higher expression in cluster 3; blue dots, genes with lower expression in cluster 3. (**B**) Pseudotime plots of selected cytokine genes in IAR CD4^+^ T cells by cluster. (**C**) Representative histogram plot of BHLHE40 expression in CD4^+^ T cells detected by flow cytometry. Cells in the top quartile of MFI were gated as BHLHE40^hi^ and cells in the bottom quartile were gated as BHLHE40^lo^. Mid refers to the middle 50th percentile of BHLHE40 expression. These gates were copied to CD4^+^ memory and CD4^+^ T cells with a cluster 3–like surface phenotype. (**D**) The average percentage of cells with the indicated BHLHE40 expression in cluster 3–like cells, CD4^+^ memory, and total CD4^+^ T cells expressed as a fraction of the total population (*n* = 5 individuals). (**E**) Representative histogram plots showing expression of GM-CSF, TNF-α, IL-2, IFN-γ, and IL-17A in BHLHE40^lo^ (red) and BHLHE40^hi^ (gray) memory CD4^+^ T cells and in BHLHE40^hi^ cluster 3–like CD4^+^ T cells (black line). (**F**) The percentage of cytokine^+^ CD4^+^ memory T cells and cluster 3–like CD4^+^ T cells with high and low BHLHE40 expression for GM-CSF, TNF-α, IL-2, IFN-γ, and IL-17A in the same individuals from **D**. Significance across groups in **D** and **F** was assessed using Friedman’s test with Benjamini-Hochberg adjustment for multiple testing. A Mann-Whitney *U* test was used for 2-group comparisons in **F**. *P < 0.05; **P < 0.01; ***P < 0.001.

We then focused our attention on the gene expression profiles of IAR CD4^+^ T cells in cluster 3. To identify markers enriched in expression in cluster 3 cells, we performed differential gene expression analysis, comparing cells in cluster 3 with all other clusters using Monocle 3 regression analysis. This analysis revealed 153 genes that were significantly upregulated (*P* < 0.05) in cluster 3 IAR CD4^+^ T cells and 184 genes that were significantly downregulated (Figure 5A and Supplemental Table 4). Notably, IAR CD4^+^ T cells from cluster 3 expressed significantly higher levels of *TNFRSF9* (CD137, *q* = 6.3 × 10^–17^), *IL2RA* (CD25, *q* = 5.9 × 10^–7^), and the transcription factor *BHLHE40* (*q* = 4.4 × 10^–14^), and significantly lower expression of *IL7R* (CD127, *q* = 7.8 × 10^–10^) (Figure 2, C–F, and Supplemental Figure 3E). The cells in cluster 3 also expressed high levels of *CD2* (Supplemental Figure 3E), *CSF2* (GM-CSF), *IL2*, *IFNG* (IFN-γ), *IL17A*, and *TNF* (Figure 5B), all cytokines reported to be regulated by BHLHE40 in T cells (29–31). We did not observe significantly different expression of genes for cytokines with tolerogenic or antiinflammatory function (e.g., *IL10*, *TGFB1*, *TGFB2*). Qualitatively similar results were obtained upon repeating the differential gene expression analysis after excluding naive-like cells in cluster 1, suggesting that differential expression of the genes in cluster 3 was primarily a property of memory-like cell clusters. We also did not note any indication of differential gene expression associated with different HLA class II alleles, as expected, since our data set was predominantly HLA DR4 positive.

We sought to independently confirm that CD4^+^ T cells with a cluster 3 phenotype express the transcription factor BHLHE40 and proinflammatory cytokines using flow cytometry. To accomplish this, we identified differentially expressed genes in cluster 3 cells that would distinguish these cells from others by flow cytometry, selecting CD137, CD2, CD25, and CD127 as the main surface markers identifying this population (Figure 5A and Supplemental Figure 3E). Since CD137 can also be expressed by Tregs, we included FOXP3 staining to further differentiate cluster 3 cells as non-Treg (FOXP3^–^). Cytokines selected for analysis included GM-CSF, TNF-α, IL-2, IFN-γ, and IL-17A, which were expressed in cluster 3 IAR CD4^+^ T cells in the scRNA-seq data and/or are regulated by BHLHE40 (32) (Figure 5B).

PBMCs from 5 established T1D patients (Supplemental Table 5) were stimulated overnight with anti-CD3/anti-CD28 beads to assess their functionality by intracellular cytokine staining in relation to BHLHE40 expression. Cluster 3–like cells were gated as CD4^+^CD45RA^–^CD45RO^+^FOXP3^–^CD2^hi^CD25^+^CD127^–^CD137^+^ (Supplemental Figure 4A). Within the CD4^+^ population, T cells in the top 75th percentile of BHLHE40 expression were compared with the cells in the bottom 25th percentile of expression to define high versus low BHLHE40 expression, respectively (Figure 5C). These percentile gates were then applied to total CD4^+^ memory T cells and cluster 3–like cell populations. We confirmed that approximately 85% of cells expressing cluster 3 markers expressed BHLHE40 at high levels, compared with approximately 60% in CD4^+^ memory cells and approximately 30% in total CD4^+^ T cells (*P* < 0.001) (Figure 5D and Supplemental Figure 4C). After overnight stimulation, we compared cytokine expression between BHLHE40^hi^ and BHLHE40^lo^ CD4^+^ memory T cells and cluster 3–like cells with high BHLHE40 expression (Supplemental Figure 4B). There were low numbers of cluster 3–like cells with low BHLHE40 expression (Supplemental Figure 4C), so comparison was made to BHLHE40^lo^ CD4^+^ memory T cells.

We detected expression of GM-CSF, TNF-α, IL-2, and IFN-γ in CD4^+^ memory T cells and cluster 3–like cells, whereas expression of IL-17A was lower (Figure 5E). Comparison of cytokine expression in relation to BHLHE40 expression in the 5 T1D patients confirmed that there was a significant difference in the percentage of cytokine-positive cells across the 3 populations (*P* < 0.01, *P* < 0.05) (Figure 5F). A significantly higher percentage of BHLHE40^hi^ CD4^+^ memory T cells and cluster 3–like cells expressed GM-CSF and TNF-α than BHLHE40^lo^ cells (Figure 5F). IL-2 expression was also increased in BHLHE40^hi^ CD4^+^ memory T cells compared with BHLHE40^lo^ cells, although there was a lower frequency of IL-2^+^ cluster 3–like cells. No significant differences were detected between individual populations for IFN-γ and IL-17A, consistent with their lower expression. These results support the notion that CD4^+^ T cells with a cluster 3–like phenotype express BHLHE40 protein and proinflammatory cytokines.

## Discussion

Analysis of changes in immune phenotypes in T1D clinical trials has revealed clues to the mechanism of action of several immunotherapies and characteristics of the response to therapy (20, 33–35). However, few studies have identified immune phenotypes at baseline (pretreatment) that can predict treatment outcome in patients with T1D, particularly among IAR T cells. Here, we analyzed rare IAR CD4^+^ T cells in PBMCs collected at baseline from alefacept- and placebo-treated new-onset T1D patients enrolled in the T1DAL clinical trial with the goal of identifying characteristics of autoreactive CD4^+^ T cells that predicted response to therapy. We identified 2 notable features at baseline that correlated with the rate of C-peptide change in alefacept-treated patients: the frequency of IAR CD4^+^ T cells with a proinflammatory phenotype and the absolute number of IAR CD4^+^ TCM cells. Both features were inversely correlated with C-peptide preservation. Neither of these features was significantly correlated with rate of C-peptide change in the placebo group, indicating that they were specific for alefacept treatment. These findings complement a previous report that baseline frequencies of antiinflammatory CD4^+^CD25^+^CD127^hi^ T cells at T1D diagnosis are correlated with a favorable response to alefacept (21) and may indicate that response to therapy is linked to the balance of proinflammatory autoreactive cells with this antiinflammatory cell population. It will be important to determine whether these measures are mutually exclusive or whether a composite biomarker of both measures is more predictive of outcome.

Overall, IAR CD4^+^ T cells had diverse phenotypes. IAR CD4^+^ T cells were primarily naive and TCM, which is consistent with antigen experience in the new-onset T1D patients. Notably, IAR CD4^+^ T cells with a TSCM phenotype were significantly increased compared with total CD4^+^ T cells and virus-reactive T cells. All Th subsets were represented, with levels of Th2 cells that were similar to those of more pathogenic Th1, Th17, and Th1/17 subsets. IAR CD4^+^ T cells had a significantly higher frequency of Th1/17–polarized cells than total CD4^+^ T cells in the same individuals and higher Th2 and Th17 cell frequencies compared with virus-reactive T cells. We cannot exclude the possibility of IAR CD4^+^ T cells with a Tfh-like phenotype since CXCR5 was not included in our flow panel. Interestingly, IAR CD4^+^ TSCM and TCM cell populations expressed more CD38 than virus-reactive T cells, which may suggest recent activation in vivo. This aligns with a previous study, which found that expression of CD38 on IAR memory CD4^+^ T cells could distinguish them from islet T cells from healthy donors (36). Importantly, nearly all IAR CD4^+^ T cells expressed CD2, the target of alefacept, ensuring their ability to be targeted by the immunotherapy.

Further dissection of the diverse phenotypes of IAR CD4^+^ T cells was achieved by examining their scRNA-seq transcript profiles, which generated a phenotypic trajectory based on a combination of maturation and activation status. Expansion of IAR CD4^+^ T cells based on shared TCR chains was detected, primarily among cells with memory transcript profiles. Four of the 5 clusters of IAR CD4^+^ T cells shared TCR chains, suggesting further activation and differentiation to effector memory cells. Notably, analysis of the individual clusters revealed that the frequency of IAR CD4^+^ T cells in cluster 3 was inversely correlated with C-peptide in the alefacept-treated individuals, but not in the placebo group. The frequency of cluster 3 cells was directly related to the frequency of IAR TCM cells, suggesting that cells with a cluster 3 transcript phenotype contributed to the association of IAR CD4^+^ TCM cells with alefacept response. Further analysis of cluster 3 cells revealed a proinflammatory phenotype characterized by expression of the transcription factor BHLHE40 and the proinflammatory cytokines GM-CSF, TNF-α, IFN-γ, IL-17A, and IL-2. We confirmed by flow cytometry that circulating CD4^+^ memory T cells from T1D patients with a similar surface phenotype expressed BHLHE40 and higher levels of GM-CSF, TNF-α, IFN-γ, and IL-17A upon activation. Thus, new-onset T1D patients with a higher frequency of proinflammatory IAR CD4^+^ T cells at baseline had a greater decline in C-peptide with alefacept treatment.

BHLHE40, also known as Bhlhb2, Dec1, and Stra13, is a member of the basic helix-loop-helix transcription factor family that binds to class B E-box DNA sequences with the consensus motif CACGTG (37). This transcription factor is of growing interest in the field of autoimmune and inflammatory diseases due to its crucial involvement in T cell activation and regulation of cytokine production in CD4^+^ T cells (29, 31, 32, 38). Recent studies have also linked BHLHE40 expression in intratumoral T cells with effective antitumor responses following immune checkpoint blockade (39, 40). Evidence from both humans and mouse models showed that BHLHE40 modulates the downregulation of IL-10 while promoting the expression of proinflammatory cytokines, such as IFN-γ and GM-CSF (29, 31, 32, 41–43). Proinflammatory CD4^+^ cells with a similar BHLHE40^+^ phenotype have been identified in the joints of patients with juvenile arthritis (44) and these cells expressed GM-CSF, TNF-α, and IFN-γ. BHLHE40 also functions in circadian clock pathways (45–47), and we cannot rule out an impact of the circadian clock on the T cell responses detected in samples in our study since blood draws were not performed at a specified time of day in the clinical trial protocol.

Our study had some limitations. The cohort of 11 alefacept and 7 placebo new-onset individuals was relatively small, and we lacked a validation cohort due to sample limitations from the T1DAL trial. This would have added statistical power to the analyses. Further studies are required to confirm whether the number of IAR CD4^+^ T cells and/or higher frequency of BHLHE40^+^ proinflammatory IAR CD4^+^ T cells at baseline can predict response to therapy targeting CD2. It is also important to note that analysis of IAR CD4^+^ T cells in the blood may not fully reflect immune regulation occurring in the pancreas. Lastly, although alefacept production has been discontinued due to the availability of other more effective therapies for psoriasis, which is the primary indication of the drug (48), other biologics targeting CD2 are currently in development for future trials in T1D or the at-risk setting.

The results of this study may have implications for the design of future clinical trials targeting CD2. The observation that higher numbers or frequencies of IAR CD4^+^ T cells were associated with poor response to alefacept raises the possibility that dosing may be inadequate to eliminate or sufficiently reduce IAR CD4^+^ T cell populations in certain individuals. Recent studies of clinical trials with rituximab or abatacept in new-onset T1D have also suggested that dosing strategies may need to be targeted to the drug pharmacodynamic and immune profiles of individual patients for optimal responses as we move toward the goal of precision medicine in T1D (49, 50). However, the correlation of IAR CD4^+^ T cell number or proinflammatory phenotype specifically in the alefacept-treated participants but not in placebo-treated participants suggests an interaction with the drug, perhaps agonist activation of proinflammatory cells or deletion of an NK-like population with regulatory activity or CD4^+^CD25^+^CD127^hi^ antiinflammatory cells (20, 21). Overall, our results suggest a role for a subset of proinflammatory IAR CD4^+^ T cells detected in peripheral blood in pancreatic dysfunction and as potential biomarkers of treatment response in clinical trials of therapies targeting the CD2 pathway. IAR CD4^+^ T cells warrant investigation for other T cell–related therapies.

## Methods

### Clinical trial and banked human samples.

Cryopreserved PBMCs from the baseline time point (pretreatment) were obtained from 18 new-onset T1D patients enrolled in the T1DAL clinical trial (ClinicalTrials.gov NCT00965458) sponsored by the Immune Tolerance Network (18, 19). The phase II randomized, double-blind, placebo-controlled trial enrolled a total of 49 participants less than 100 days from T1D diagnosis: 33 assigned to the alefacept arm and 16 to the placebo arm. Patients received weekly injections of drug or placebo for two 12-week courses and were followed for 24 months. Of the 18 participants in the current study, 11 were treated with alefacept and 7 were treated with placebo. Patient characteristics are summarized in Table 1. The rate of C-peptide change for each individual over 24 months was estimated as exponential decay using a random effects model of log(C-peptide 2-hour AUC) values, as previously described (50, 51). All participants had at least one DRB1*04, DRB1*03, or DQB1*03 high-risk allele (only 2-digit HLA genotype data were available for this study). We performed additional validation of our findings using cryopreserved PBMCs from established T1D patients from the Benaroya Research Institute Registry and Repository. All samples were tested in a blinded manner.

### Isolation of IAR T cells.

IAR CD4^+^ T cells were isolated from cryopreserved PBMCs using a CD154 activation assay, as previously described (22, 23). Briefly, PBMCs were stimulated for 14 hours in the presence of 1 μg/mL anti-CD40 antibody (Miltenyi Biotec, clone HB14) with either a vehicle control (DMSO), positive control viral peptides (Peptivator CMV pp65, Peptivator AdV5 Hexon purchased from Miltenyi Biotec and MP8 57–76 KGILGFVFTLTVPSERGLQR and MP54 97–116 VKLYRKLKREITFHGAKEIS influenza A peptides), or a 35-peptide pool from the islet proteins GAD65, IGRP, ZnT8, IA-2, PPI, and Ins B that comprise immunodominant epitopes recognized by CD4^+^ T cells in T1D patients in the context of HLA DRB1*0401, DRB1*0301, and DQ8 (Supplemental Table 1). Following stimulation, cells were stained with PE-coupled anti-CD154 antibody and the activated CD154^+^ T cells were enriched using anti-PE magnetic beads (Miltenyi Biotec). Cells were then surface stained using fluorophore-tagged antibodies specific for CD4, CD8, CD14, CD19, CD56, CD69, CD45RA, CCR7, CD95, CCR4, CXCR3, CCR6, PD-1, TIGIT, and CD2 for flow cytometry analysis. Antibody details are listed in Supplemental Table 2. Live CD4^+^CD154^+^CD69^+^ activated cells from the islet-peptide-stimulated culture were flow sorted based on gating set to the DMSO vehicle control (Figure 1A and Supplemental Figure 1). Sorting and flow cytometry acquisition were performed with a BD FACSAria Fusion cell sorter. Cells were index-sorted into a 96-well plate containing 5 μL/well reaction buffer from the SMART-Seq v4 Ultra Low Input RNA Kit (Takara Bio) for subsequent library preparation. The frequency of IAR CD4^+^ T cells or viral antigen–reactive CD4^+^ T cells per million total CD4^+^ T cells was calculated in relation to a pre-enrichment sample using the following formula: (number of enriched IAR CD4^+^ T cells × 10^6^)/(number of CD4^+^ T cells in pre-enrichment sample × dilution factor).

### scRNA-seq and analysis.

Sorted IAR CD4^+^ T cells were subjected to cDNA synthesis and preamplification, and sequencing libraries were generated using a NexteraXT DNA sample preparation kit with dual indexes (Illumina) as previously described (22). Barcoded single-cell libraries were pooled and sequenced with single-index 58-bp reads on a HiSeq 2500 System (Illumina) to a target depth of 1.25 million reads per cell. We used the MiXCR R package to identify productive TCR α and β chain rearrangements. TCR chain comparisons between cells were made based on perfect nucleotide matching for the recombined V-J or V-D-J junction sequence from the second cysteine residue (position 104) to the J-phenylalanine or J-tryptophan residue (position 118); a chain was considered expanded if it was detected in at least 2 cells. Comparisons of TCR junctions to the databases VDJdb (26) and McPAS (27) were made using the junction amino acid sequence. Transcript analysis was performed using the Monocle 3 (25) package. Profiles were batch corrected (52) for cellular detection rate (53). Cell profiles were clustered (54) and subjected to dimensionality reduction using UMAP (55). Pseudotime analysis as implemented in Monocle 3 was performed as described previously (56), setting a seed for the pseudorandom number generator to maximize the reproducibility of clustering. Clustering was also repeated multiple times to assess reproducibility of clustering. Cell clusters were annotated by mapping reference PBMC populations to the IAR CD4^+^ T cell trajectory using the FindTransferAnchors and the MapQuery functions in Seurat (57). Genes defining clusters were determined using the top_marker function in Monocle 3. Identification of differentially expressed genes in a single cluster compared to all other clusters was determined using the fit_models regression analysis function in Monocle 3. The fit_models function fits a generalized linear model for each gene in a cell data set.

### Flow cytometry analysis.

Supervised analysis of flow cytometry data from enriched antigen-reactive CD4^+^ T cells was performed using FlowJo software v10.8.1 (Tree Star) to identify T cell subsets (Th1, Th2, Th1/Th17, Th17), maturation stages (naive, TSCM, TCM, TEM), activation and inhibitory receptor expression (CD38, PD-1, TIGIT), and expression of CD2 on islet- or virus-reactive CD4^+^ T cells as gated in Supplemental Figure 1. Flow cytometry of total CD4^+^ T cells was performed using the pre-enrichment sample from either the islet- or viral peptide–stimulated cultures. Intracellular cytokine staining on bulk CD4^+^ T cells was performed using cryopreserved PBMCs from individuals with established T1D. Cells were thawed, rested, and stimulated for 18 hours with Immunocult CD3/CD28 T cell activator cocktail diluted 1:80 (STEMCELL Technologies). Then, cells were further activated with 50 ng/mL PMA (Sigma-Aldrich) and 500 ng/mL ionomycin (Sigma-Aldrich) in the presence of 1 μg/mL Brefeldin A (BioLegend) and 1 μg/mL monensin (BD Biosciences) for 4 hours. Cells were stained with Live/Dead Blue (Invitrogen) followed by fluorescently tagged antibodies specific for extracellular markers, including CD3, CD4, CD8, CD19, CD14, CD56, CD45RA, CD45RO, CCR7, CD95, CD127, CD137, PD-1, TIGIT, CD25, CD2, and CD27. Cells were then fixed and permeabilized (eBioscience intracellular fixation and permeabilization buffer set) and stained for intracellular transcription factors (BHLEH40 and FOXP3) and cytokines (IFN-γ, IL-2, IL-17A, GM-CSF, and TNF-α). Antibodies are detailed in Supplemental Table 2. Flow cytometry was performed with a Cytek Aurora spectral cytometer and analyzed using FlowJo. Samples were gated as live, dump negative (CD14^–^, CD19^–^, CD8^–^, CD56^–^), CD3^+^, CD4^+^, CD45RO^+^, CD45RA^–^, FOXP3^–^, CD2^hi^, CD25^+^, CD127^–^, and CD137^+^, as shown in Supplemental Figure 4A. Within the CD4^+^ population, BHLHE40 expression levels were defined by identification of the 25th and the 75th percentiles of the BHLHE40 MFI using the FlowJo percentile calculation function, where cells at or below the 25th percentile were considered to have low expression for the transcription factor and cells at or above the 75th percentile were considered to have high expression (Figure 5A). These gates were then applied to the memory and cluster 3–like cells. Finally, manual gating for intracellular cytokine expression was based on a no-stimulation (no Immunocult/no PMA and ionomycin) control (Supplemental Figure 4B).

### Statistics.

Statistical tests were performed using the R programming language or GraphPad Prism version 9. Wilcoxon’s signed-rank test was used to assess differences in paired group comparisons and Mann-Whitney *U* tests were used to analyze unpaired, 2-group comparisons. Differences across cells expressing high and low BHLHE40 levels were determined using Friedman’s test. A Kolmogorov-Smirnov test was used for comparing the distribution of cells with expanded TCRs between clusters. Spearman’s *r* correlation tests were performed to assess correlations of nonparametric variables. An FDR-adjusted *P* value of less than 0.1 was used to define differential gene expression. The specific test used to derive each *P* value is listed in the figure legends. *P* values were adjusted for multiple testing using the Benjamini-Hochberg test correction (58) and adjusted *P* values of less than 0.05 were considered significant.

### Study approval.

The study was approved by the Benaroya Research Institute’s Institutional Review Board, protocols 10024 and 3041700. All participants provided written informed consent upon enrollment in the study.

### Data availability.

All data and analyses from this study are available from the ITN TrialShare public website (https://www.itntrialshare.org/project/home/begin.view) and the NCBI Gene Expression Omnibus repository (GEO GSE182870). Supporting data for graphs are included in the Supporting Data Values Excel file. R code for analysis is deposited in GitHub (https://github.com/BenaroyaResearch/Islet-autoreactive-CD4-T-cells-are-linked-with-response-to-alefacept-in-type-1-diabetes.git).

## Author contributions

KC, PSL, EB, ES, and GTN designed the study. EB, JC, VHG, and MGR performed experiments and prepared data. EB, AKH, HAD, PSL, and KC performed analyses of data, interpretation, and figure generation. ES and GTN provided T1DAL samples, meta data, and interpretation. CS and CJG recruited T1D patients and provided banked PBMC samples. EB, PSL, and KC wrote the manuscript with contributions from the other authors.

## Supplementary Material

Supplemental data

Supplemental tables 1-5

Supporting data values

## Figures and Tables

**Table 1:**
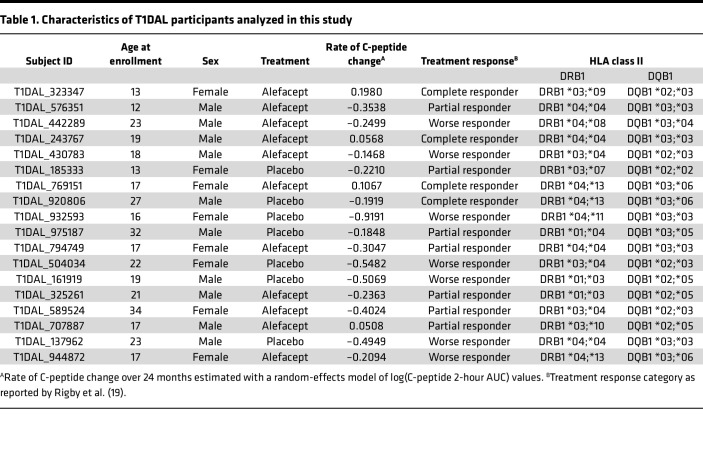
Characteristics of T1DAL participants analyzed in this study

## Version 1 09/26/2023

In-Press Preview

## Version 2 11/08/2023

Electronic publication
