# Supplementary material for: Islet-autoreactive CD4+ T cells are linked with response to alefacept in type 1 diabetes
Source: JCI Insight. 2023 Nov 8;8(21):e167881. doi: 10.1172/jci.insight.167881 (PMC10721267; doi:10.1172/jci.insight.167881)
Supplement: Supplemental data [file jciinsight-8-167881-s157.pdf]

Supplemental Figure 1

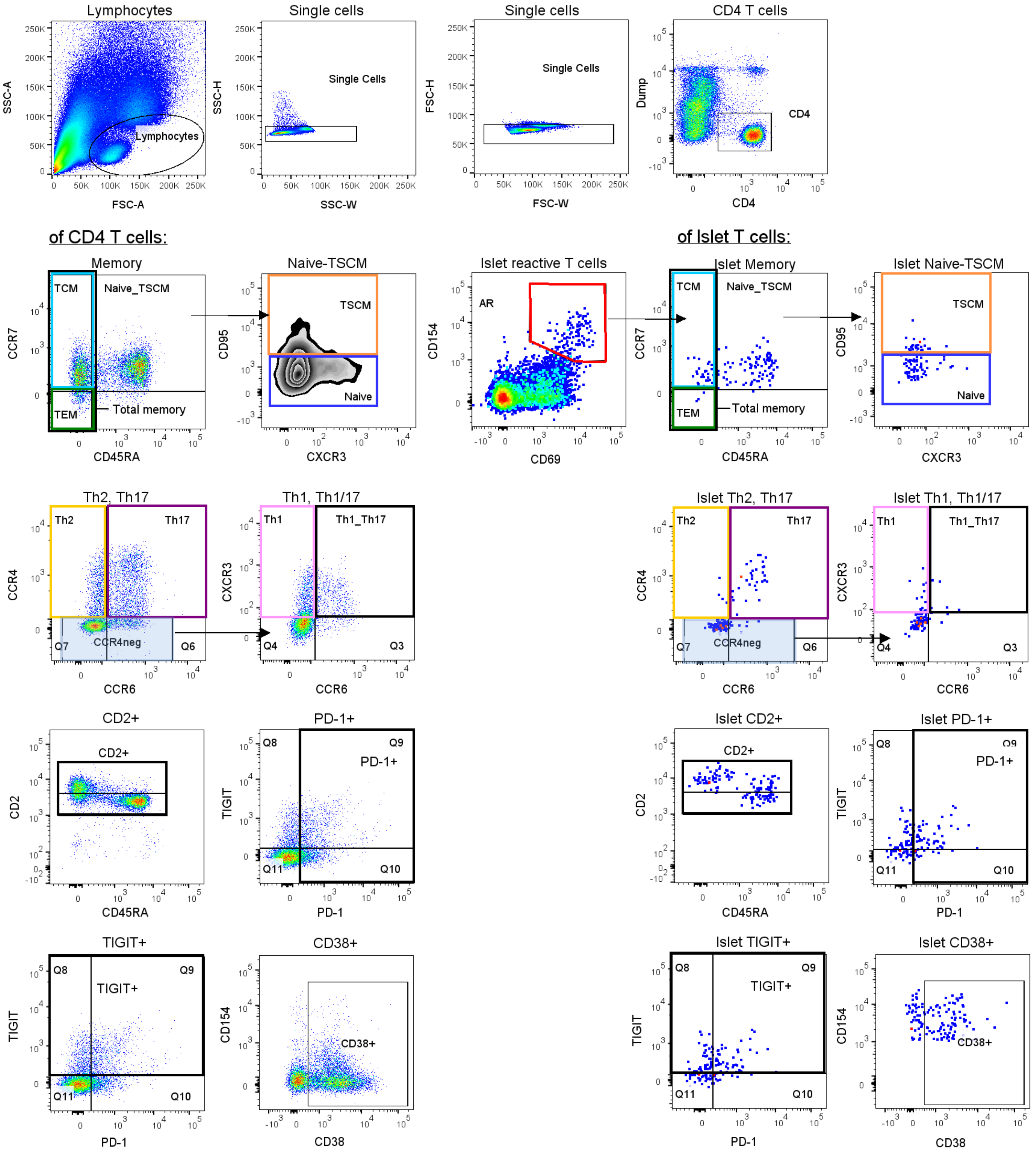

Supplemental Figure 1. Flow cytometric gating strategy for antigen reactive CD4 T cell populations in a PBMC sample stimulated for 14h with islet peptides (subject T1DAL-243767). Gates for naïve and memory populations, T cell helper subsets, activation and inhibitory receptors were set using total CD4 T cells and were then applied to the islet antigen reactive population.

Supplemental Figure 2

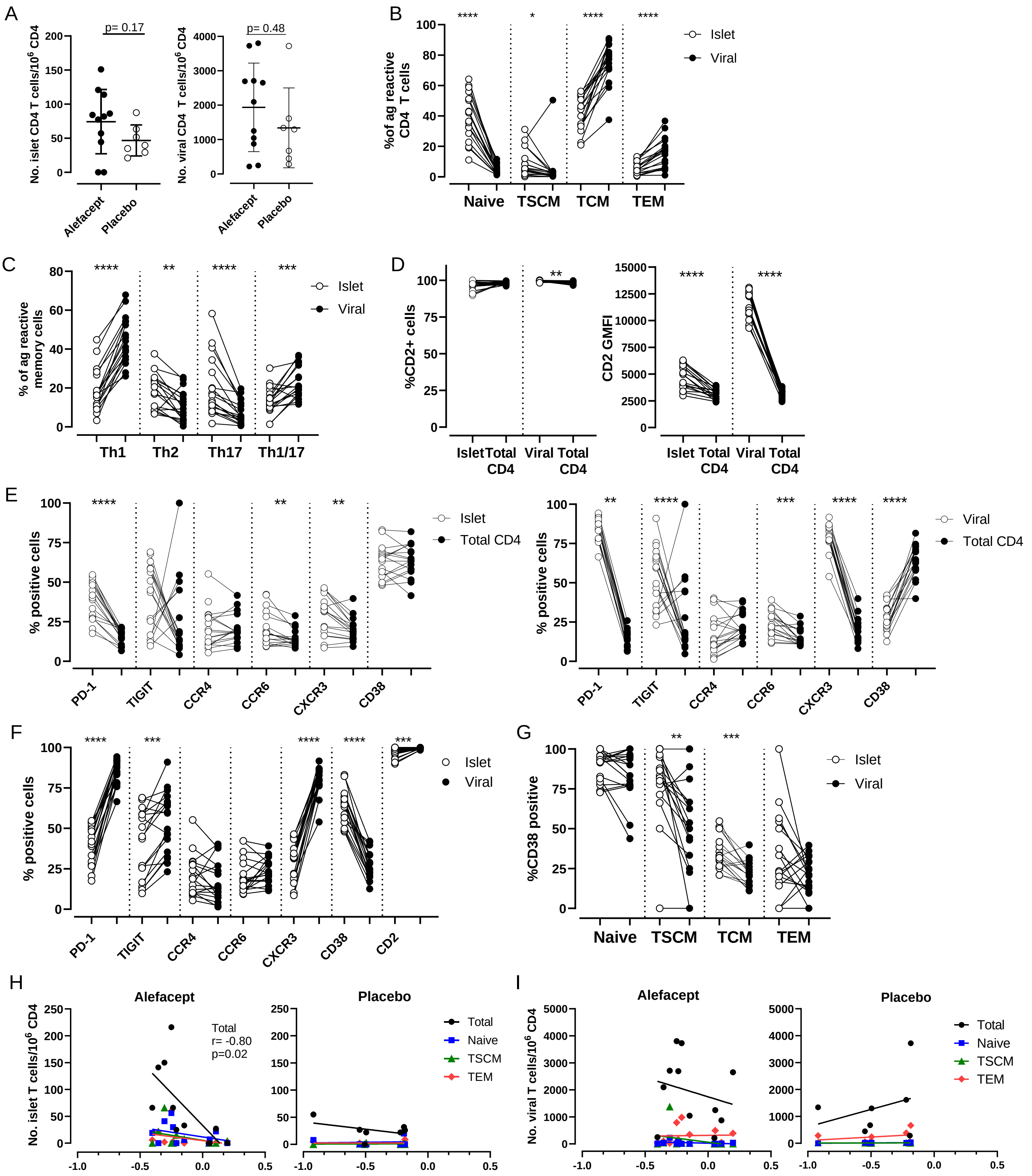

Supplemental Figure 2

J

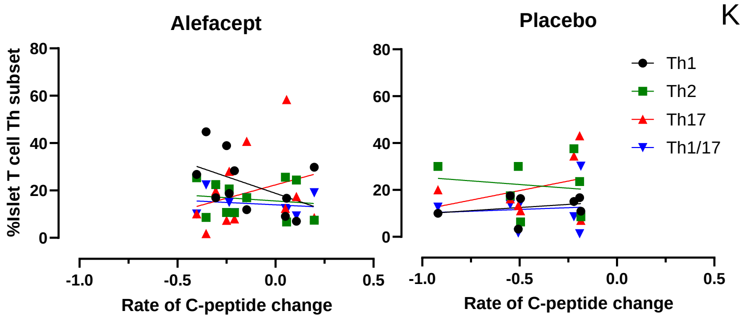

K

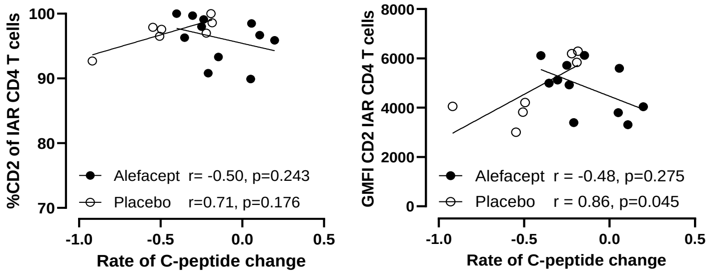

Supplemental Figure 2. (**A**) The number of IAR or viral reactive CD4 T cells per million total CD4 T cells in alefacept- (n=11) versus placebo-treated (n=7) subjects. Each symbol represents a unique subject. Graphs depict mean +/- SD. Significance was assessed using a Mann Whitney U test. (**B**) The frequency of naïve and memory populations in IAR CD4 T cells compared with viral reactive T cells per sample (n=18). (**C**) The frequency of Th subsets in IAR CD4 memory T cells compared with viral reactive T memory cells per sample (n=18). (**D**) The expression of CD2 on enriched IAR and viral antigen reactive CD4 T cells versus bulk CD4 T cells from the pre-enrichment sample from the same cultures, shown as the percent CD2+ antigen reactive or bulk CD4 T cells (left) and as CD2 GMFI (right) from all subjects (n=18). (**E**) The expression of individual surface markers on enriched IAR (left) or viral (right) reactive CD4 T cells compared with bulk CD4 T cells from the pre-enrichment sample from the same cultures (n=18 subjects) shown as the percent positive cells of the respective population. (**F**) Expression of surface markers on enriched IAR CD4 T cells compared with viral reactive T cells per sample (n=18). (**G**) Expression of CD38 on enriched IAR CD4 T cells compared with viral reactive T cells naïve and memory populations (n=18). (**H**) Correlation between the frequency of total, naïve, TSCM, and TEM IAR CD4 T cells per subject with the rate of C-peptide change in alefacept- (n=11) and placebo-treated subjects (n=7). Significant differences are noted in the graphs. (**I**) Correlation between the frequency of total, naïve, TSCM, and TEM viral reactive CD4 T cells per subject with the rate of C-peptide change in alefacept- and placebo-treated subjects. (**J**) Correlation between the frequency of memory IAR CD4 T cells with the indicated Th phenotypes with the rate of C-peptide change in alefacept (n=11) and placebo groups (n=7). (**K**) Correlation between the frequency of CD2+ IAR CD4 T cells (left) or CD2 GMFI on IAR CD4 T cells (right) with the rate of C-peptide change in alefacept (n=11) and placebo groups (n=7). Significant differences between populations in the graphs in B-G were determined using a Wilcoxon matched-pairs signed rank test with Benjamini-Hochberg adjustment and are indicated by asterisks, **P* <0.05, ***P* <0.01, ****P* <0.001, *****P* <0.0001. Correlations in the graphs in H-K were performed using a Spearman rho test and P values were adjusted using Benjamini-Hochberg adjustment.

Supplemental Figure 3

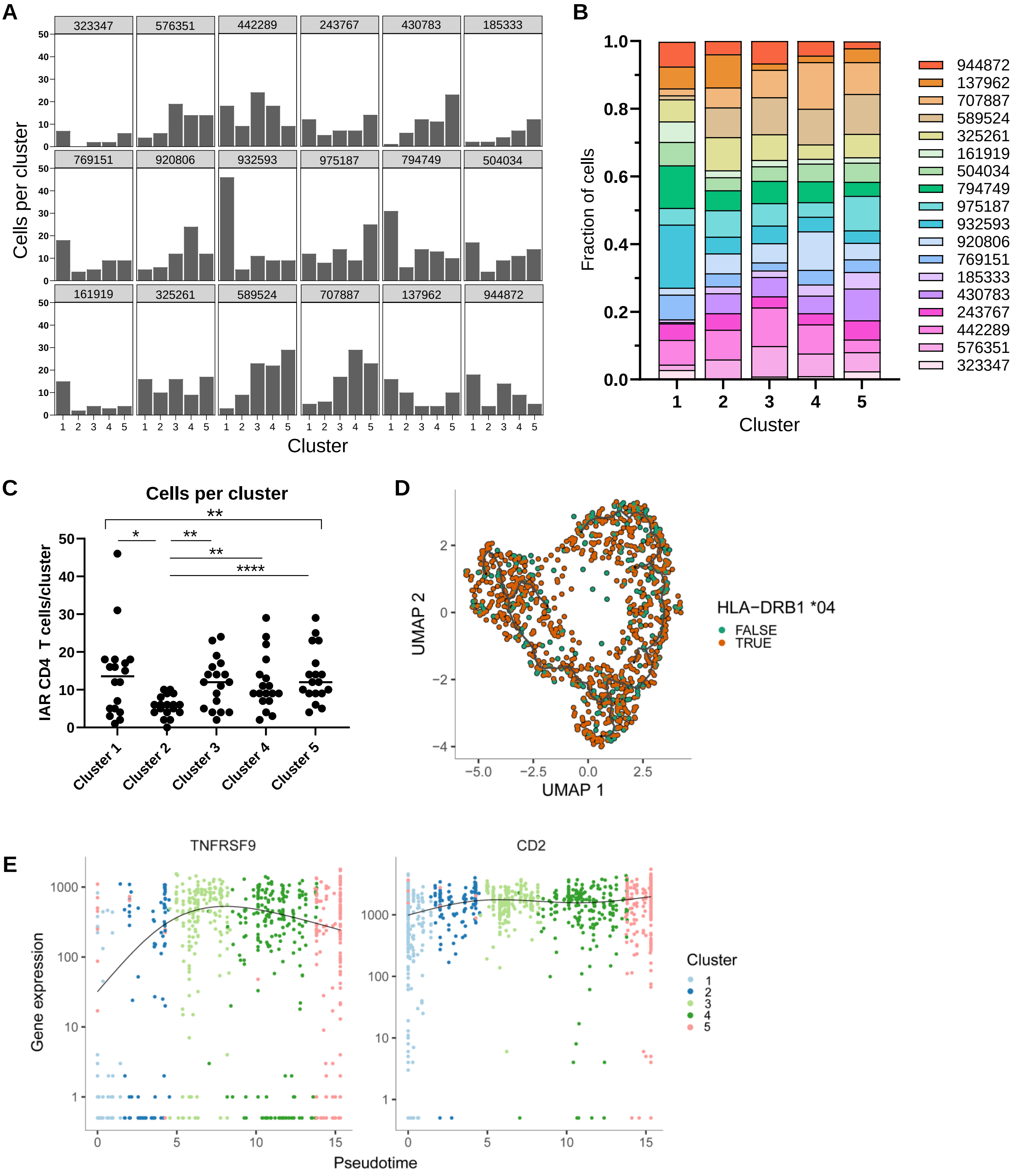

Supplemental Figure 3. (**A**) The distribution of IAR CD4 T cells in Monocle Clusters 1-5 in each T1DAL participant in this study. Subject IDs are indicated at the top of each graph. (**B**) The fraction of each Monocle cluster composed of cells from each donor in the study. (**C**) Comparison of the distributions of IAR CD4 T cells between Monocle clusters. Variance across clusters was determined using a Kruskal Wallis test; two-group comparisons were made using a Mann Whitney U test. **P* <0.05, ***P* <0.01, *****P* <0.0001. (**D**) Monocle 3 pseudotime trajectory of IAR CD4 T cells colored by donor HLA DRB1*04 status, showing no bias in cell clustering by HLA. (**D**) Pseudotime plots showing high levels of transcripts for TNFRSF9 (CD137) and CD2 in Monocle Cluster 3.

Supplemental Figure 4

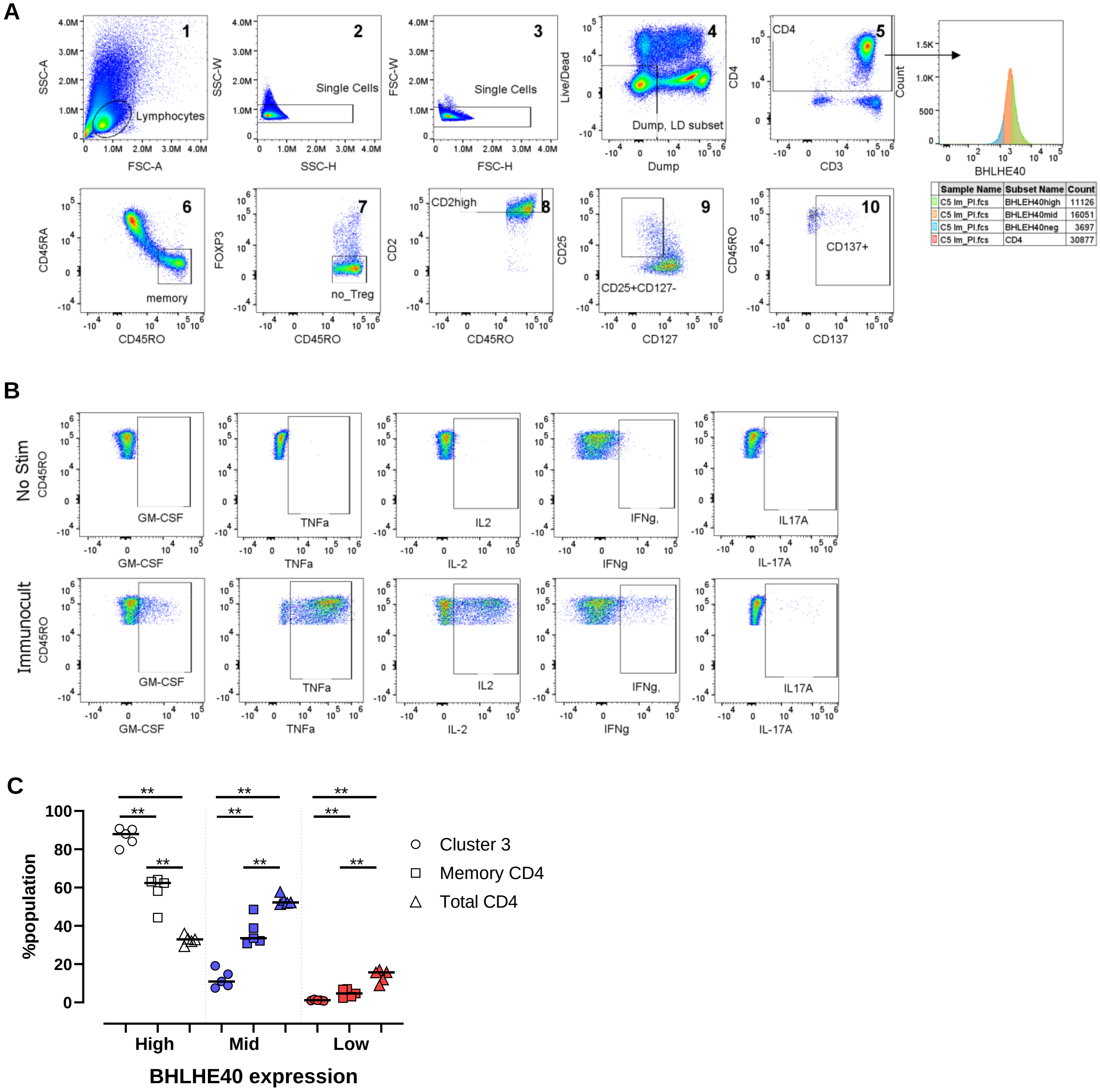

Supplemental Figure 4. Identification of Cluster 3-like CD4 T cells by flow cytometry and analysis of intracellular cytokine expression. (**A**) Gating strategy to identify Cluster 3-like CD4 T cells expressing low and high levels of BHLHE40. (**B**) Gates for intracellular cytokine expression were set using CD4 memory T cells from cultures that were not stimulated with anti-CD3/anti-CD28 antibodies (Immunocult) and copied onto Cluster 3-like cells. (**C**) Two-way comparisons of the frequency of cells expressing high, mid, or low levels of BHLHE40 amongst CD4 T cells with Cluster 3 phenotype, CD4 memory T cells, and total CD4 T cells as shown in Figure 4D, n=5 T1D subjects. Significance was assessed using Mann Whitney U tests with Benjamini-Hochberg adjustment for multiple testing, $^{**}P < 0.01$.
